# Spike-Driven Neighborhood Attention for Vision Transformers

**DOI:** 10.3390/e28101130

**Published:** 2026-10-15

**Authors:** Marzieh Hassanshahi Varposhti, Mahyar Shahsavari, Marcel van Gerven

**Affiliations:** Department of Machine Learning and Neural Computing, Donders Institute for Brain, Cognition and Behaviour, Radboud University, 6500HB Nijmegen, The Netherlandsmarcel.vangerven@donders.ru.nl (M.v.G.)

**Keywords:** Spiking Neural Networks, spiking transformers, Spike-Driven Neighborhood Attention

## Abstract

Spiking Neural Networks (SNNs) promise ultra-low-power, event-driven computation; however, current efforts to integrate self-attention into spiking transformers predominantly rely on global attention mechanisms, introducing growing token complexity when scaling and diluting discriminative feature representations. We introduce Spike-Driven Neighborhood Attention (SDNA) over the full k×k neighborhood in two forms: Pairwise SDNA, which gates each query–neighbor interaction before aggregating, and Linear SDNA, which exploits the absence of a softmax in spiking attention to pool the key–value interaction over the window and gate it once, evaluating the spiking gate per position rather than per query–neighbor pair. Both operators are placed in a fully local hierarchical backbone that increases the receptive field without any global attention layer. Across static and neuromorphic benchmarks the two operators are competitive with global spike-driven transformers at comparable or lower parameter counts. Pairwise SDNA achieves the highest accuracy among the compared spike-driven transformers on CIFAR-10 at 97.24%, while Linear SDNA achieves the highest accuracy on N-Caltech101 at 89.65%. These results show that competitive performance can be achieved using a fully local hierarchical attention design, without requiring an explicit global-attention stage.

## 1. Introduction

Spiking Neural Networks have emerged as biologically inspired alternatives to conventional Artificial Neural Networks (ANNs), offering low-power, event-driven computation [1,2,3,4,5] with a natural match to neuromorphic hardware [6]. Grounded in the dynamics of biological neurons [7], they have been combined with deep architectures to exploit spatial and temporal structure and applied across a range of tasks [8,9,10,11]. Convolutional spiking models, however, struggle to capture long-range dependencies without substantial memory and energy overhead [12,13]. Transformers, developed for language [14,15,16] and extended to vision [17,18,19,20], address this through attention, though at a significant compute and memory cost [21,22]. Spiking transformers were introduced to combine the low-energy, event-driven nature of SNNs with the representational power of attention [23,24,25,26].

Most spiking transformers adopt *global* attention, in which every token interacts with every other. Beyond its quadratic cost, dense global interaction can dilute the response in the task-relevant regions that carry the discriminative signal, which has motivated a shift toward local attention in the non-spiking setting [27,28,29]. In the spiking domain this direction remains largely unexplored. A recent local spiking attention mechanism [30] restricts attention through sparse dilated sampling, which lowers cost but discards most of the neighborhood and can lose the fine local structure a full window would retain.

The spike-driven transformer (SDT) [24] already exploits one consequence of removing the softmax. Without a normalization over the attended set, the key–value interaction can be aggregated first and the query applied afterwards, which yields a linear-cost global operator [24]. This reordering has so far been used only for global attention, where a single aggregate is shared by all queries. In the local setting the same freedom takes a different and unexploited form, because each query attends to its own window and no single aggregate can be shared.

We bring this reordering to local attention. We first construct the direct spiking realization of neighborhood attention, Pairwise SDNA, in which each query is scored against every key in its k×k neighborhood, the score is gated by a spiking neuron, and the gated values are summed. Because the spiking gate is pointwise rather than a normalization over the window, its placement is not structurally constrained to precede aggregation. This permits us to define a second operator in which the neighborhood is pooled before gating. Both operators attend over the full k×k neighborhood rather than a sparsely sampled subset of it, so the fine local structure a window carries is preserved. We place both operators in a hierarchical spiking backbone that applies the same local operator at every stage while progressively reducing spatial resolution. Through composition across stages and dilated neighborhoods, the effective receptive field expands without requiring a global-attention layer.

Our contributions are summarized as follows: First, we introduce two Spike-Driven Neighborhood Attention operators over the full k×k neighborhood, Pairwise SDNA and Linear SDNA, and show that the absence of a softmax in spiking neighborhood attention allows us to formulate a pooled neighborhood operator in which the spiking gate is applied after window aggregation, without introducing the softmax approximation or kernel feature mapping required by many conventional linear-attention formulations. Second, we show that the reordered operator, Linear SDNA, evaluates the spiking gate once per position rather than once per query–neighbor pair, and we characterize what this placement of the gate costs in selectivity and saves in computation. Third, we build a fully local hierarchical spiking transformer in which the same neighborhood operator is applied at every stage, so a global receptive field is reached by composition rather than by any global attention layer. Finally, to demonstrate the effectiveness of our approach, we perform extensive experiments on static and neuromorphic benchmarks, including CIFAR-10, CIFAR-100, N-Caltech101, DVS128-Gesture and ImageNet, together with ablations that isolate the effect of gate placement, gate calibration, and membrane time constant.

## 2. Related Works

### 2.1. Spiking Transformers

Transformer architectures have been adapted to SNNs to give them content-dependent, adaptive feature aggregation, which convolution’s fixed kernels cannot provide. The difficulty is that self-attention is also the most expensive component to make spike-driven, its cost grows with the number of tokens, and softmax normalization has no spike-form equivalent. The work below can be read as successive attempts to retain the benefit of attention while reducing what it costs, first by simplifying the computation, then by changing the abstraction, and finally by restricting its spatial scope. Spikformer [23] introduced the first fully spiking transformer, replacing softmax attention with spiking self-attention (SSA) that operates directly on binary spike tensors. Removing the softmax leaves an attention map computed from spike-form queries and keys, but the operator remains global, every token attends to every other, at a cost quadratic in the number of tokens.

SDT [24] simplified the arithmetic of that operator. Since the product of two spike tensors reduces to a logical AND, SDT replaces the matrix multiplications of SSA with element-wise Hadamard products and a column-wise sum, so that attention performs only mask-and-accumulate operations and is genuinely spike-driven on neuromorphic hardware. The scope stays global, but the cost becomes linear in the token count; single key–value aggregate is formed once and shared across all queries.

QKFormer [31] changed the abstraction of attention rather than its arithmetic. Its Q-K token attention computes an importance score from the query and key alone, with no value–side interaction and no pairwise attention map, giving linear complexity in the token count. This operator is used in the early stages, where tokens are many and channels few, while the later stages revert to spiking self-attention. The whole is arranged as a hierarchical backbone whose token count decreases and embedding dimension increases from stage to stage, placing the cheapest attention where the tokens are most numerous.

These operators restrict what attention computes while leaving its scope global. LSFormer [30] instead restricts where attention looks, introducing a local structure-aware spiking self-attention that samples keys and values along horizontal and vertical dilated strips and assigns different dilation rates to different channel groups to recover multi-scale receptive fields. Its efficiency comes from attending to fewer sampled positions rather than from the structure of the local window itself.

Wherever these operators aggregate over a set of query–key interactions, whether globally as in SSA and SDT or locally as in LSFormer, the spiking nonlinearity is applied to each interaction before that aggregation. The ordering is inherited from softmax attention, where normalizing over the attended set requires the nonlinearity to act on the assembled scores. A spiking gate carries no such requirement. It is pointwise, and nothing binds it to the per-pair scores. Whether the gate must precede aggregation, and what is gained by moving it, is the question this work takes up.

### 2.2. Efficient Attention Mechanisms

Global self-attention computes an interaction between every pair of tokens, so its cost and memory grow quadratically with the sequence length. Restricting attention has therefore become the dominant route to efficiency in vision transformers. Swin [28] partitions the feature map into non-overlapping windows and computes attention within each, reducing cost to linear in the token count for a fixed window size, and recovers cross-window information by shifting the partition between consecutive blocks. Neighborhood attention [27] replaces partitioning with a sliding window that each query attends to the k×k window of its nearest neighbors, so the windows overlap and move with the query. This preserves translation equivariance, unlike the shifted-partition scheme, requires no pixel shifts to grow the receptive field, and reduces to dot-product self-attention when the window reaches the size of the feature map. Both localize attention by restricting which keys a query may attend to, while leaving the internal structure of the operator unchanged. In standard softmax attention, normalization is performed jointly over the attended query–key scores. Consequently, the individual pairwise scores must be formed before the normalized weights can be applied to the values, constraining the computation to pairwise scoring, normalization, and value aggregation. The key–value interaction therefore cannot simply be pooled before the attention weights are formed, because the normalized weights depend on the individual query–key scores. Efficient non-spiking attention methods that avoid this quadratic formulation generally modify the attention operator, for example, by replacing the conventional softmax-based similarity with a kernel or feature-map formulation that permits reassociation of the computation.

Spiking attention removes the softmax outright [23], since it has no spike-form equivalent, and replaces it with a pointwise spiking gate. The absence of normalization across the attended neighborhood means that the gate is not structurally required to process all pairwise scores before aggregation. This permits an alternative formulation in which the key–value interaction is pooled over the neighborhood before the spiking gate is applied, with the query applied afterwards. Importantly, this does not make the resulting operator (Linear SDNA) mathematically equivalent to the pairwise formulation, because the spiking gate is nonlinear. Rather, the softmax-free setting permits this alternative gate placement without introducing an additional softmax approximation or kernel feature mapping. The present work takes up this possibility and examines what it costs and what it saves.

## 3. Materials and Methods

This section develops two Spike-Driven Neighborhood-Attention operators. Neighborhood attention has been brought to spiking networks before [30], though in a different architecture and a different formulation from ours. We first construct the direct spiking realization, Pairwise SDNA, which gates every query–neighbor pair (Section 3.2). We then consider an alternative placement in which, because the spiking gate is pointwise rather than a normalization over the window, the neighborhood is aggregated before the gate is applied. This does not preserve the exact Pairwise SDNA function because the spiking gate is nonlinear; rather, it defines a distinct softmax-free operator, Linear SDNA, that removes a factor of $k^{2}$ from the gating cost.

### 3.1. Preliminaries

In a spiking network, the activations passed between layers are spike events, not real numbers. Under multi-spike neurons an activation is a small non-negative integer; under binary neurons it is either 0 or 1. The energy-relevant quantity is the number of synaptic operations (SOPs), that is, spike-triggered accumulations. As established in the spike-driven transformer line [24], a product of two spikes reduces to a logical AND, so the only arithmetic spike-driven layer performs is a spike-gated accumulation, in which a spike triggers one accumulation and silence triggers nothing. The data path has no multipliers and no subtractors. We treat this event-driven sparsity as the property the operator must preserve, and throughout this paper we call an operator *spike-driven* only when every elementary operation it performs is a spike-gated accumulation.

#### 3.1.1. Neuron Model

The base unit is the leaky integrate-and-fire (LIF) neuron [1], written in the iterative form used for direct training. At physical time-step *t*, the membrane potential charges, fires, and resets according to

(1) $$\begin{array}{cc} U[t] & =\beta \;H[t-1]+(1-\beta )X[t], \end{array}$$

(2) $$\begin{array}{cc} S[t] & =\mathrm{Hea}\left(U\left[t\right]-V_{\mathrm{th}}\right), \end{array}$$

(3) $$\begin{array}{cc} H[t] & =U\left[t\right]-V_{\mathrm{th}}\;S\left[t\right], \end{array}$$

where X[t] is the input from the preceding layer, U[t] is the membrane potential after charging but before firing, H[t] is the potential after firing, leak term β=1−1/τ with τ the membrane time constant, $V_{\mathrm{th}}$ is the threshold, S[t] is the output spike, and Hea(·) is the Heaviside step function. The leak β<1 carries information across time-steps. The non-leaky integrate-and-fire variant replaces (1) with U[t]=H[t−1]+X[t], integrating the unscaled input current without decay. Equation (3) is the soft reset, which subtracts the threshold on firing and is the reset we use; a hard reset, which instead clears the potential to a fixed value, is the alternative.

We build on the integer-firing neuron of the Spike Firing Approximation (SFA) method [32]. A binary spike discards the magnitude of a neuron’s response, collapsing strong and marginal activations to the same single spike, whereas the integer code retains that magnitude and preserves the representational precision that dense inputs and attention scores depend on. At inference the integer activation is unrolled into an equivalent binary spike train over *D* virtual sub-steps, exactly, so the deployed network is purely binary and spike-driven. We write SN(·) for this neuron applied element-wise, which is the gate used in the operators below. Static models use it at a single physical time-step, while event models use a leaky form that integrates evidence across physical time-steps with a per-dataset time constant τ. The integer fire function, the virtual-step construction, and the two temporal regimes are given in Appendix A.1.

#### 3.1.2. Spiking Transformer Backbone

The operator sits inside a hierarchical spiking-transformer backbone. We write *N* for the number of spatial tokens at a stage, *C* for the channel count, *T* for the number of physical time-steps, and *k* for the square neighborhood kernel size, so each query attends over a k×k window. The query, key and value spike tensors are *Q*, *K*, *V*; $Q_{i}$ is the query at position *i*, and $K_{j}$, $V_{j}$ are the key and value at a neighbor *j*. The set of $k^{2}$ positions in the window centered on *i* is W(i). The windows may be dilated, changing the membership of W(i) but leaving the analysis below unchanged. We write ⊙ for the channel-wise Hadamard product which treats channels independently.

Restricting attention to a local window forces a choice. A neighborhood operator needs a different window sum at every position, and the only way to compute these overlapping sums with spike-gated accumulation alone is to re-accumulate each window directly, at $O(k^{2})$ cost per position; the kernel-independent alternative, an integral image, relies on dense subtraction and is not spike-driven. We keep the spike-drivenness and accept the $O(k^{2})$ cost, which is what a per-neighbor operator already pays; Appendix A.2 gives the full argument. Our contribution, as detailed in Section 3.2, is that within this spike-driven branch, one design choice removes a factor of $k^{2}$ from both the number of spiking-gate evaluations and attention memory, at a small and measured cost in selectivity.

Once window accumulation is fixed, one decision settles the operator, namely where to place the spiking gate relative to the window sum. The gate is the nonlinearity that turns the accumulated evidence into spikes. It can act on each neighbor before the sum, or once on the pooled sum, and these two placements give the two operators below. The two formulations represent different points between shared regional aggregation and per-neighbor gating. The two are instantiated by the same registered model with identical parameters; only the forward computation changes.

### 3.2. Spike-Driven Neighborhood Attention

**Figure 1:**
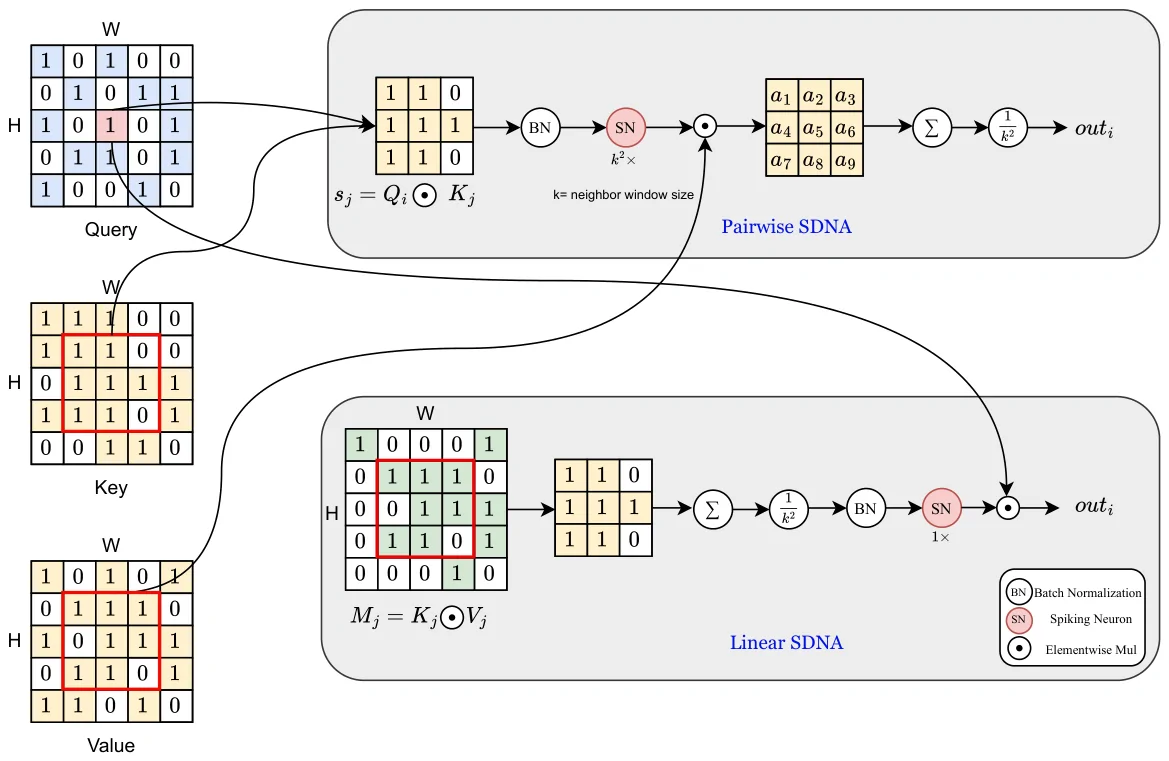
Overview of the two Spike-Driven Neighborhood Attention operators. **Top:** Pairwise SDNA scores each query ($Q_{i}$) against every neighboring key ($K_{j}$) by element-wise product, gates each score with a spiking neuron (SN), sums the gated values over the window, and scales the result by the window size. **Bottom:** Linear SDNA first pools the key–value product (*M*) over the neighboring window and scales it, then gates the pooled result once and applies the query afterward, so the gate is evaluated once per position rather than once per neighbor. The batch normalization on the gate input, shown in both operators, is used only on the neuromorphic models, where it re-centers the pooled coincidence so the gate does not fall silent on sparse event data.

We now introduce Spike-Driven Neighborhood Attention (SDNA), illustrated in Figure 1. Pairwise SDNA is the direct spiking realization of neighborhood attention (Figure 1, top). It scores each query–neighbor pair (4), gates each score with the spiking neuron (5), and sums the gated values over the window(6):

(4) $$\begin{array}{cc} s_{ij} & =Q_{i}⊙K_{j}, \end{array}$$

(5) $$\begin{array}{cc} a_{ij} & =\mathrm{SN}\;\left(s_{ij}\right), \end{array}$$

(6) $$\begin{array}{cc} {\mathrm{out}}_{i} & =\frac{1}{k^{2}}\sum_{j\in W(i)}a_{ij}⊙V_{j}. \end{array}$$

This provides the direct pairwise formulation, and it keeps per-neighbor selectivity, since it can respond to a single strong neighbor while retaining neighbor-specific selectivity. It is also the operator against which the reformulation is measured below. The costs follow from Equations (4)–(6). The operator materializes per-neighbor score and attention tensors whose size grows with $k^{2}$, it gates $Nk^{2}C$ values (one spiking-gate evaluation for every neighbor), and its attention memory scales as $O(Nk^{2})$.

The linear SDNA operator (Figure 1, bottom) computes the key–value coincidence once per position, pools it over the window, gates the pooled result a single time, and re-weights by the query:

(7) $$\begin{array}{cc} M_{j} & =K_{j}⊙V_{j}, \end{array}$$

(8) $$\begin{array}{cc} L_{i} & =\frac{1}{k^{2}}\sum_{j\in W(i)}M_{j}, \end{array}$$

(9) $$\begin{array}{cc} {\mathrm{out}}_{i} & =Q_{i}⊙\mathrm{SN}\;\left(L_{i}\right). \end{array}$$

The coincidence $M_{j}$ in Equation (7) is computed once per position and reused by every window that contains *j*. The pooling in Equation (8) forms the spike-driven window-accumulation branch. The gate then acts once on the pooled value. Note that the query enters after the gate, modulating the gated regional aggregate rather than a per-neighbor score. The factor $1/k^{2}$ keeps the gate input in a bounded band as *k* grows. We call this operator Linear SDNA because the spiking gate is evaluated once per position, so the number of gate evaluations is linear in the token count and independent of the window size, where the pairwise form evaluates the gate for every query–neighbor pair and scales with the window.

Because the gate is a pointwise spiking neuron rather than a softmax normalized over the window, the SN nonlinearity is not tied to the per-pair scores, and it can be applied either before or after the window sum. The two placements are therefore both admissible, but they are not equivalent. Gating each neighbor and then summing is not the same function as summing and then gating, since the gate is nonlinear. The two operators differ only in this order, and the resulting difference in what they compute is measured in the results. The order of gating and summation contrasts as

(10) $$\underset{\mathrm{Pairwise}:\;\mathrm{gate}-\mathrm{then}-\mathrm{sum}}{\underset{︸}{\sum_{j}\mathrm{SN}\left(s_{j}\right)}}\;\mathrm{vs}.\;\underset{\mathrm{Linear}:\;\mathrm{sum}-\mathrm{then}-\mathrm{gate}}{\underset{︸}{\mathrm{SN}\;\left(\sum_{j}M_{j}\right)}},$$

and in where the query enters, at the score or at the final product. Three cost properties follow from this one change. Linear SDNA performs $k^{2}$ times fewer spiking-gate evaluations, one per position (NC) rather than one per neighbor ($Nk^{2}C$), and that count does not grow with *k*. Its attention-map memory is O(N) and independent of *k*, against $O(Nk^{2})$ for pairwise, so the neighborhood can be widened without a memory penalty. And the key–value coincidence is computed once per position and reused by every window that contains it, rather than recomputed for each query–neighbor pair, which lowers the constant factor at the same asymptotic cost $O(Nk^{2}D)$.

Sum-then-gate cannot tell which neighbor carried the evidence. It sees only the regional aggregate $\sum_{j}M_{j}$, where gate-then-sum makes a separate decision per neighbor. Pairwise SDNA can respond to a single discriminative neighbor and identify it. In contrast, Linear SDNA pools weak evidence into one robust quantity and gives up the per-neighbor detail.

#### 3.2.1. Neighborhood Positional Encoding

The window in Equations (4) and (8) is symmetric and per channel, so on its own the operator cannot tell a neighbor above the query from one below it, and some form of positional information must be supplied. The standard mechanism in windowed attention is a relative position bias (RPB) [28,33], a learnable bias indexed by the query–neighbor offset and added to the score of each pair. RPB applies naturally to Pairwise SDNA, where a per-pair score exists (Equation (4)). It does not transfer to Linear SDNA. There, the neighbor contributions are summed into a single aggregate $L_{i}$ before the gate acts (Equation (8)), which collapses the relative offsets that RPB indexes, so no per-pair bias can be attached. A relative-position signal could, in principle, be reintroduced by weighting neighbors before pooling, but this is no longer RPB.

We therefore supply position through a Conditional Positional Encoding (CPE) [34], which both operators can use. CPE is a depthwise convolution (DWConv) per-block k×k followed by batch normalization (BN), added residually to the spike tensor before each attention block,

(11) $$x\leftarrow x+\mathrm{BN}\left({\mathrm{DWConv}}_{k\times k}\left(x\right)\right).$$

Because it acts on the token map rather than on per-pair scores, CPE is independent of the operator and applies unchanged to both Pairwise and Linear SDNA. Over binary spikes, the depthwise convolution is accumulation-only, and the encoding is small. It is the architecture’s main positional signal, and Linear SDNA relies on it alone, while Pairwise SDNA could additionally use RPB.

#### 3.2.2. Neighbor Gating

In neuromorphic datasets, the gate requires one adjustment. The input of the pooled window (Equations (4) and (8)) is the average of spike coincidences over the window, and on sparse event streams this average sits close to zero, so the gate rarely reaches its firing threshold and falls largely silent. We therefore normalize the gate input on the neuromorphic models before gating,

(12) $${\hat{S}}_{i},{\hat{B}}_{i}=\mathrm{BN}\left(S_{i}\right),\mathrm{BN}\left(L_{i}\right)$$

and the gate acts on ${\hat{S}}_{i}$ and ${\hat{L}}_{i}$ for the pairwise and linear models, respectively. This centers the pooled coincidence across the firing threshold and restores gate activity; its effect is analyzed in Section 4. The static models, whose dense inputs keep the gate active, gate $L_{i}$ directly and do not use this normalization.

### 3.3. Architecture Overview

QKFormer applies different attention operators at different depths, using a token-level attention (QKTA) which forms an importance mask from queries and keys alone, in the early token-rich stages, and full spiking self-attention (SSA) in the final stage. We depart from this and at all stages, including the earliest and highest-resolution, use the same local operator, the Spike-Driven Neighborhood Attention block of Section 3.2. No stage forms a global token-to-token map. This is made viable by two properties acting together. First, the SDNA operator’s cost is linear in the number of tokens and independent of the grid extent, so it stays affordable on the large early grids where a global operator would not be. Second, the hierarchy itself supplies global reach, since a fixed-size window covers a small fraction of an early, fine grid but a large fraction of a late, coarse one, so the effective receptive field grows by composition across stages rather than by any single global layer. The network is therefore uniformly local, yet globally expressive.

**Figure 2:**
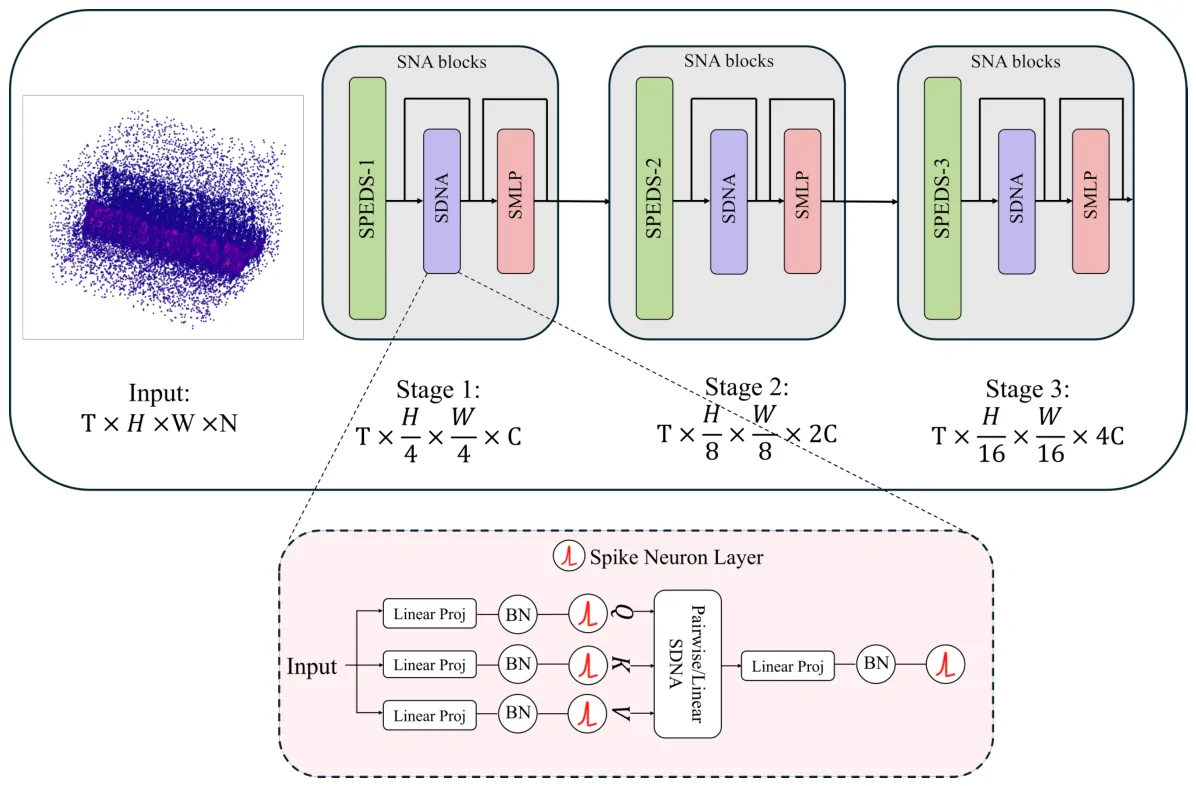
Hierarchical architecture used for SDNA operators; each stage applies the SDNA operator at a fixed window with per-stage dilation.

We adopt the hierarchical spiking transformer backbone of QKFormer [31], illustrated in Figure 2, comprising a spiking patch-embedding module (SPEDS) that also performs down-sampling between stages, a sequence of stages operating at decreasing spatial resolution and increasing channel width, and a classification head that is grouped over space and time. The SPEDS modules and the membrane-shortcut residual convention are taken from that design unchanged, and we describe them only briefly in the following. The input tensor has *N* channels (N=2 polarity channels for event data, N=3 for RGB static images); every tensor exchanged between components after the first SPEDS is a spike tensor, so the network is accumulate-only, and the neuron model is the one defined in Section 3.1. Our architectural contribution is not the backbone, but what fills its attention layers, described next.

Each stage is a stack of identical blocks, and each block applies three residual operations in sequence,

(13) $$\begin{array}{cc} x & \leftarrow x+\mathrm{CPE}(x), \end{array}$$

(14) $$\begin{array}{cc} x & \leftarrow x+\mathrm{SDNA}(x), \end{array}$$

(15) $$\begin{array}{cc} x & \leftarrow x+\mathrm{SMLP}(x). \end{array}$$

CPE is the depthwise convolution of Equation (11) for positioning the local windows. The SDNA operator projects the block input to spiking queries, keys, and values with linear projections, restricts mixing to the dilated window around each position, and projects the result back; its interior, where the spiking gate sits relative to the window sum, is the subject of Section 3.2 and is this paper’s main contribution. The SMLP is the Spiking Multilayer Perceptron used for channel mixing in spike-driven transformers [23]; it is identical across the operator variants we compare. All three residual additions are spike-tensor additions, following the membrane-shortcut convention of the backbone rather than inserting a neuron on the shortcut path.

Every stage uses the same 3×3 window, so the number of attended positions is fixed across the hierarchy, and only the dilation varies, decreasing with depth so that each stage’s effective reach stays commensurate with its grid.

The remaining components (the SPEDS patch embedding and downsampling modules and the classification head) are taken from the backbone [31] and are detailed in Appendix A.3.

## 4. Results

In this section, we evaluate the two SDNA approaches on static image benchmarks, including CIFAR-10/100 [35] and ImageNet [36], as well as the event-driven neuromorphic benchmarks N-Caltech101 [37] and DVS128-Gesture [38].

### 4.1. Experimental Setup

All SDNA models share the following configuration. We train with AdamW and a cosine learning-rate schedule with linear warmup, label smoothing of 0.1, and automatic mixed precision. Every stage uses a 3×3 neighborhood window with per-channel attention, conditional positional encoding, and integer-firing neurons. Each model is trained with five random seeds, and we report the mean and standard deviation, except on ImageNet, where results are from a single run. Table 1 lists the settings that differ between experiments. Unless otherwise stated, results for competing methods are taken from their corresponding original publications or published comparative literature; only the SDNA results are obtained from our own experiments.

### 4.2. CIFAR Results

On the static CIFAR benchmarks (Table 2), Pairwise SDNA attains the highest CIFAR-10 accuracy in the comparison at 97.24±0.08%, above LSFormer [30] (96.73±0.07) and QKFormer [31] (96.18), with Linear SDNA close behind at 96.87±0.09%; both exceed most prior methods on this dataset at fewer parameters (6.76 M against 9.32 M for Spikformer and 9.50 M for LSFormer). On CIFAR-100, Pairwise SDNA reaches 81.73±0.22%, compared with 81.87±0.16% for STMixer and 82.00±0.04% for LSFormer, placing its mean accuracy 0.14 and 0.27 percentage points below these methods, respectively. It exceeds QKFormer (81.15%), while Linear SDNA reaches 80.98±0.13%.

### 4.3. ImageNet Results

We evaluated the operators at scale on ImageNet-1K at 224×224. Beyond the common settings of Section 1, the ImageNet models use a three-stage backbone of ten blocks with per-stage dilation of 7, 5, and 4, as well as a base learning rate of $6\cdot 10^{-4}$, scaled to an effective batch size of 512 over four GPUs. We use augmentation techniques including RandAugment [43] and random erasing [44], following DeiT. Results are reported for a single run. Table 3 shows that both SDNA operators scale well on ImageNet. We additionally include QKFormer, whose hierarchical backbone forms the basis of our architecture but which employs different attention mechanisms across stages, using QK token attention in the earlier stages and spiking self-attention in the later stage. QKFormer reaches 78.80% and 82.04% accuracy for its HST-10-384 and HST-10-512 configurations, respectively. In comparison, Pairwise and Linear SDNA reach 77.94%/77.67% at 384 dimensions and 78.22%/77.93% at 512 dimensions. While QKFormer achieves higher ImageNet accuracy, the SDNA results show that strong large-scale recognition performance can be obtained using a uniform local attention operator throughout the hierarchy, without an explicit global-attention stage.

Table 3 further reports the theoretical arithmetic energy of the compared ImageNet models. At the 384-dimensional configuration, Pairwise and Linear SDNA require 16.38 and 16.33 mJ per image while reaching 77.94% and 77.67% Top-1 accuracy, respectively. QKFormer provides a stronger absolute ImageNet operating point, achieving higher accuracy with lower reported arithmetic energy. However, SDNA addresses a different architectural objective. Both variants employ the same neighborhood-based attention principle throughout the hierarchy, restricting interactions to bounded local regions rather than introducing an explicit global-attention stage. The results therefore show that competitive large-scale recognition performance can be maintained with a uniformly local spike-driven attention design. The similar whole-model energy of Pairwise and Linear SDNA is mainly due to the shared weighted computation, while Linear SDNA retains its reduced gate-evaluation and attention-memory requirements.

### 4.4. Neuromorphic Benchmarks

We compared the two SDNA operators against prior spiking transformers on the neuromorphic benchmarks in Table 4. On N-Caltech101, Linear SDNA reaches the highest accuracy in the comparison, exceeding the global-attention models SDT [24] and Spikformer [23] by +3.35 and +6.05, respectively, and improving on QKFormer [31] by +2.41. It also exceeds LSFormer [30], the closest local spiking-attention method, by +2.05. Pairwise SDNA is likewise competitive, above all prior methods on this dataset. On DVS128-Gesture, where accuracies are saturated near the ceiling, both operators reach 98.89% on average over multiple seeds, ahead of Spikformer (+0.59), QKFormer (+0.29), and LSFormer (+0.29), and within 0.41 points of SDT, which is highest at 99.30%. Both operators achieve these results with fewer parameters than Spikformer and SDT, although QKFormer uses fewer parameters than either SDNA variant.

In contrast to the static datasets where Pairwise SDNA outperforms Linear SDNA, for neuromorphic datasets, Linear outperforms Pairwise. This suggests that for sparse event streams Linear is the stronger of the two, consistent with the pooling in Linear SDNA aiding aggregation where activity is sparse and costs little where it is dense.

### 4.5. Theoretical Arithmetic Energy Consumption

The theoretical arithmetic energy of Pairwise and Linear SDNA is estimated from their activity-dependent synaptic operations. Following prior work on spike-driven networks, we distinguish between multiply-and-accumulate (MAC) operations for weighted layers receiving continuous-valued inputs and accumulate-only (AC) operations for weighted layers receiving spike-valued inputs. For a spike-driven weighted layer *l*, the number of synaptic operations is estimated as

(16) $$SOP^{l}=f^{l}\times T\times FLOPs^{l},$$

where $f^{l}$ denotes the input firing rate, *T* is the number of physical timesteps, and $FLOPs^{l}$ denotes the corresponding dense operation count. For integer-firing neurons, the integer activation represents the number of equivalent binary spike events over the *D* virtual sub-steps. The measured integer spike count therefore already incorporates the virtual spike activity and is not multiplied by *D* again. The SDNA attention operations are also counted as spike-driven synaptic operations. We denote their activity-dependent cost by $SOP_{\mathrm{attn}}$. The total spike-driven operation count is

(17) $$SOP_{\mathrm{total}}=\sum_{l\in A}SOP^{l}+SOP_{\mathrm{attn}},$$

where A denotes weighted layers receiving spike-valued inputs. Weighted layers receiving continuous-valued inputs form the set M, with

(18) $$MAC_{\mathrm{total}}=\sum_{l\in M}MAC^{l}.$$

Using the 45 nm arithmetic-energy estimates [47], $E_{\mathrm{AC}}=0.9$ pJ and $E_{\mathrm{MAC}}=4.6$ pJ, the theoretical arithmetic energy is

(19) $$E_{\mathrm{SDNA}}=E_{\mathrm{AC}}\;SOP_{\mathrm{total}}+E_{\mathrm{MAC}}\;MAC_{\mathrm{total}}.$$

This estimate represents theoretical arithmetic energy rather than measured hardware power and excludes memory access, neuron-state updates, control logic, and data movement. We applied the process described above to measure the ImageNet energy consumption shown in Table 3.

In addition, Table 5 shows distinct firing patterns for the two SDNA formulations. Linear SDNA exhibits higher activity in the *K*, *V*, and attention-gate paths. In particular, its higher gate firing rate is consistent with the aggregation-before-gating formulation, where neighborhood responses are accumulated before being passed through the nonlinear gate. This denser aggregated signal can produce higher gate activity despite the substantially smaller number of gate evaluations. Consequently, the structural reduction in gate evaluations does not translate proportionally into whole-network energy reduction.

### 4.6. Analysis and Ablations

We next analyze the differences between the two operators and the factors governing their behavior on event data. We first isolate the cost of the reordering, measuring how the memory and throughput of the two operators scale with the neighborhood size. We then examine two factors specific to the neuromorphic setting, the membrane time constant and the calibration of the attention gate, both of which act on the same underlying quantity, the rate at which the network fires. Together, these establish where the efficiency of Linear SDNA comes from and why the event models require the choices made in Section 3.

#### 4.6.1. Efficiency of the Reordering

**Figure 3:**
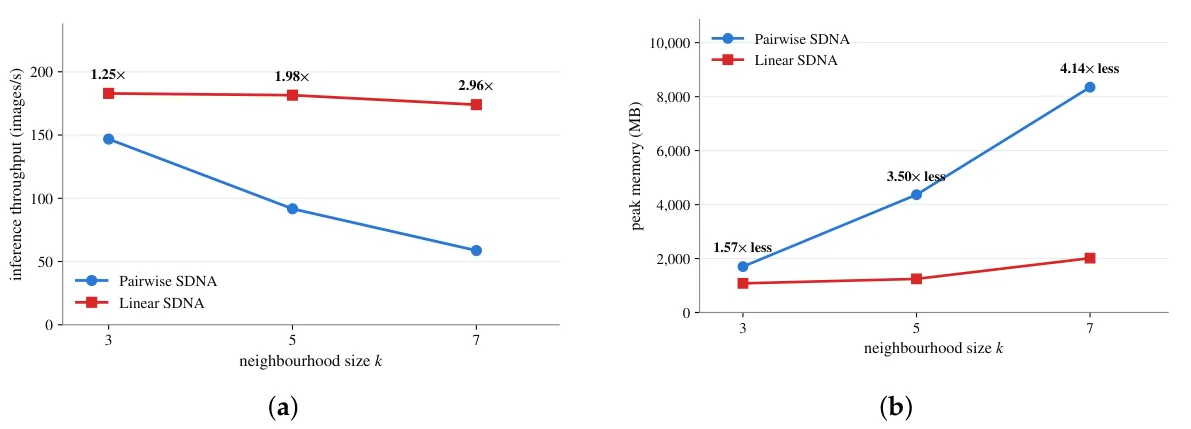
Efficiency of the two operators as the neighborhood size k grows; all else fixed. (**a**) Inference throughput. (**b**) Peak memory.

Figure 3 compares the two operators directly as the neighborhood grows, with every other factor held fixed. The pair of neighbors incurs a cost that increases with *k*. At the window k=3 used in our models, the difference is already present at 1.57× lower memory and 1.25× higher throughput, and it increases steadily as the neighborhood expands, reaching 4.14× lower memory and 2.96× higher throughput at k=7. The two operators are therefore not interchangeable in cost even though they attend over the same window. The reordering that leaves accuracy essentially unchanged is what makes the larger neighborhoods affordable, since Linear SDNA sustains them at close to constant cost while Pairwise SDNA does not.

#### 4.6.2. Batch Normalization Effect on Gate Channels

**Figure 4:**
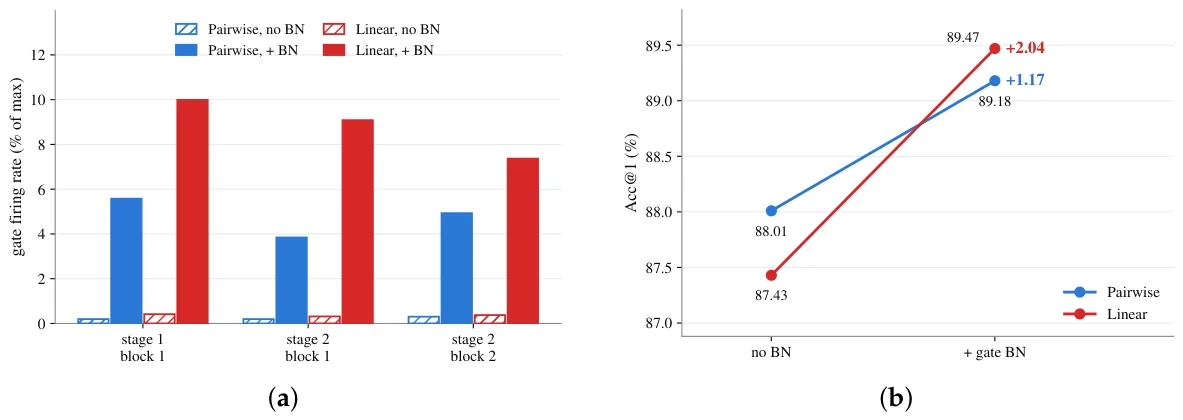
Effect of gate calibration on N-Caltech101 (single seed, ablation configuration). (**a**) Gate firing rate, as a percentage of its maximum, across the three attention blocks, with and without the normalization. (**b**) Adding the channel-wise normalization on the gate input for both operators.

The attention gate consumes an unnormalized product of two spike quantities, and on the sparse event data this product concentrates close to zero, so the fixed firing threshold is rarely reached and the gate is largely silent. Adding channel-wise normalization to the gate input re-expands this distribution across the threshold. Figure 4a shows the effect directly: without the normalization the gate barely fires in any block, and adding it lifts the gate firing rate across all three. Figure 4b shows the accuracy that follows, +1.17 for Pairwise and +2.04 for Linear. The larger gain for Linear SDNA is consistent with its stronger dependence on the gate because it applies a single gate to the pooled window rather than one gate per neighbor; a starved gate removes proportionally more of its signal.

#### 4.6.3. Effect of the Membrane Time Constant

The leaky integer neuron used on the event benchmarks integrates over the physical time-steps with the update mem[t]=βmem[t−1]+(1−β)x[t], where a single scalar τ sets the decay β=1−1/τ. Because the leak acts only across physical steps, it applies only to the event models; the static models run at a single physical step, so τ has no effect (Appendix A.1).

Table 6 sweeps τ homogeneously across all spiking nodes, with every other setting fixed at the reported configuration. Three observations follow. First, the optimum is consistent across all four curves, over both datasets and both operators, peaking at τ=1.5. That the two operators agree matters, since it means τ is a property of the backbone and the data rather than something re-tuned per operator, so the operator comparison is not confounded by a per-operator choice of τ. Second, the effect is large on N-Caltech101 and the sweep spans roughly five accuracy points, larger than the gap between the two operators. Third, the optimum is interior and the falloff is asymmetric, with accuracy degrading gently below τ=1.5 and steeply above it.

The mechanism behind the sweep is the network’s firing rate. Training on N-Caltech101 across different τ, higher τ produces markedly lower firing and lower accuracy. As shown in the table, raising τ to 3 starves the gate and costs about five accuracy points. Firing rate tracks accuracy across most of the range but does not fully determine it. The highest firing model (τ = 1.25) is not the most accurate, so more spiking is not monotonically better. The performance degradation below the optimum is consistent with reduced temporal integration. As τ falls, β decreases and the membrane retains less history, which on event data, where the signal is spread across frames, costs more than the added drive returns.

## 5. Discussion

In this paper, we introduced Spike-Driven Neighborhood Attention and studied where the spiking gate should sit relative to the neighboring window. The direct form, Pairwise SDNA, gates each query–neighbor interaction before aggregating, while the reordered form, Linear SDNA, exploits the absence of a softmax in spiking attention to pool the key–value interaction first and gates it once. The central finding is that this reordering incurs only a small accuracy difference while changing the cost of the operator. Restricting attention to local neighborhoods and reducing the gating to a single evaluation per position therefore preserves competitive representational performance. Across static and neuromorphic benchmarks the two operators remain competitive with global spike-driven attention, and on N-Caltech101 and CIFAR-10 they exceed it.

A single mechanism accounts for much of what we observe, namely the rate at which the network and its attention gate fire. The membrane time constant controls this rate directly. As it increases, the network fires progressively less and activation sparsity rises, and beyond an interior optimum the model is starved of spikes and loses accuracy, so the best operating point is one of moderate rather than maximal or minimal firing. Gate calibration influences the same quantity through a different mechanism. Without it, the unnormalized gate input on sparse event data sits below the firing threshold, and the gate falls largely silent; restoring its activity through normalization recovers the lost accuracy. That two independent interventions, one on the neuron time constant and one on the gate input, move accuracy through the same firing-rate channel indicates that spike availability, rather than any single architectural choice, is the binding constraint on these operators. The ImageNet analysis further shows that the operator-level simplifications of Linear SDNA, including substantially fewer gate evaluations, do not directly translate into proportional whole-network energy savings, because firing activity and shared weighted computation remain important contributors. More broadly, the results indicate that both Pairwise and Linear SDNA can maintain competitive large-scale recognition performance using a uniformly local attention formulation, without requiring an explicit global-attention stage.

Our results place these operators within a line of spiking transformers that reduce the cost of attention in different ways. Spikformer [23] and the spike-driven transformer [24] retain full global attention, in which every token interacts with every other, while QKFormer [31] reduces this cost by forming a token-importance mask from queries and keys alone. These methods retain a global attention scope, whereas our experiments show that competitive performance can also be achieved using a fully local hierarchy. By restricting attention to a local neighborhood, SDNA avoids diluting the response across the entire feature map, and the proposed operators remain competitive with these global models while surpassing them on N-Caltech101 and CIFAR-10, which indicates that the discriminative signal for these tasks is already present within a local window. The reduction in computational complexity does not necessarily imply a corresponding loss in recognition accuracy. In SDNA, locality acts as an inductive bias that concentrates attention on spatially relevant neighboring features, while the hierarchical backbone progressively enlarges the effective receptive field across stages. As a result, the model can reduce unnecessary long-range interactions without necessarily reducing the discriminative information available for classification. The one prior spiking method to take a local route, LSFormer [30], reaches locality differently, sampling sparse dilated strips within a columnar backbone rather than attending over the full neighborhood in a hierarchy. Attending to the complete k×k window, our operators remain competitive with LSFormer across the evaluated benchmarks and exceed it on N-Caltech101 and CIFAR-10.

Two limitations qualify these results. The first concerns where the efficiency of the operators can be realized. The reduction we obtain is in the number of spiking-gate evaluations and in attention memory, quantities that fall regardless of the platform, but converting that reduction into wall-clock speed depends on the hardware. Current GPUs are optimized for large dense matrix multiplication and reward neither the accumulate-only nature of spike–spike interaction nor the fine-grained, locally windowed access pattern of neighborhood attention, so on a GPU the operation-count advantage of the local operator is not reflected in runtime. The saving is therefore best read as a property of the computation rather than a measured speed-up on general-purpose hardware.

The second limitation is one of measurement. Our strongest results are on the neuromorphic benchmarks, where the datasets are small and the test sets limited in size, so seed-to-seed variance is non-negligible and the accuracy differences between close configurations are not always individually separable. The time-constant sweep in particular was run at a single seed and used for selection rather than for statistical comparison between adjacent values, and the reported accuracies, while averaged over several seeds, carry standard deviations that overlap for the nearest competing methods on some datasets. These results should therefore be read as evidence of a consistent trend and a competitive operating point rather than of a precisely resolved ranking.

Both limitations point toward the same line of future work. A spike-driven local operator maps naturally onto hardware built for event-driven accumulation and fine-grained spatial access rather than dense matrix products, and a dedicated accelerator could exploit the sparsity and locality that a general-purpose GPU leaves unused, realizing the operation-count advantage as an end-to-end latency and energy gain. Alongside this, evaluation on larger benchmarks would tighten the variance associated with the relatively small neuromorphic datasets used in the current study, while future work could investigate the applicability of SDNA to more complex downstream vision tasks, such as object detection and semantic segmentation.

## Figures and Tables

**Table 1:** Per-group training and model settings for static image benchmarks (CIFAR-10, CIFAR-100), the large-scale ImageNet dataset and the neuromorphic benchmarks (N-Caltech101, DVS128-Gesture).

Setting	CIFAR	ImageNet	Neuromorphic
Input	32×32	224×224	224×224/128×128
Patch size per stage	1, 2, 4	4, 8, 16	8, 16
Stages/blocks	3/1, 1, 2	3/1, 2, 7	2/1, 1
Embed dims	96→192→384	96→192→384	128→256
Dilation per stage	4,3,2	7,5,4	4,3/3,2
Epochs	400	200	300/200
Batch	64	512	16
Peak LR	0.001	0.0012	0.001
Weight decay	0.06	0.05	0.06
MLP ratio	4	4	1

**Table 2:** Comparison of accuracy and parameter count on CIFAR10/100. Bold numbers are the best performance.

Method	Architecture	Param	Time Step	Accuracy	
				CIFAR-10	CIFAR-100
Spikformer [23]	Spikformer-4-384	9.32	4	95.51	78.21
SDT [24]	S-Trans-2–512	10.28	4	95.60	80.02
STAtten [39]	S-Trans-2–512	9.32	4	96.03	79.85
QKFormer [31]	HST-4-384	6.74	4	96.18	81.15
SWformer [40]	SWformer-4-384	7.51	4	96.10	79.30
SAFormer [41]	SAFormer-4-384	9.32	4	95.80	79.07
STMixer [42]	STMixer-4-384-32	8.29	4	96.01 ± 0.11	81.87 ± 0.16
LSFormer [30]	LSFormer-4-384	9.50	4	96.73 ± 0.07	**82.00 ± 0.04**
Pairwise SDNA (ours)	SDNA-4-384	6.76	4	**97.24 ± 0.08**	81.73 ± 0.22
Linear SDNA (ours)	SDNA-4-384	6.79	4	96.87 ± 0.09	80.98 ± 0.13

**Table 3:** Comparison of accuracy and parameter count on ImageNet. NR=Not reported.Bold numbers are the best performance.

Method	Architecture	Param (M)	Time-Step	Energy (mJ)	Accuracy (%)
Vision Transformer [17]	ViT-B/16(ANN)	86.59	1	254.84	77.90
Spikformer [23]	Spikformer-8–384	16.81	4	7.73	70.24
	Spikformer-8–512	29.68	4	11.58	73.38
SDT [24]	S-Transfor-8-384	16.81	4	3.90	72.28
	S-Transfor-8-512	29.68	4	4.50	74.57
STAtten [39]	S-Transfor-8-512	29.68	4	NR	76.18
	S-Transfor-8-768	66.34	4	12.36	78.11
QKFormer [31]	HST-10-384	16.47	4	15.13	78.80
	HST-10-512	29.08	4	21.99	**82.04**
Pairwise SDNA (ours)	SDNA-10-384	16.59	4	16.38	77.94
	SDNA-10-512	29.27	4	28.79	78.22
Linear SDNA (ours)	SDNA-10-384	16.59	4	16.33	77.67
	SDNA-10-512	29.27	4	28.71	77.93

**Table 4:** Comparison of accuracy and parameter count on N-Caltech101 and DVS128-Gesture. * Reconstructed from the architecture reported in the original publication. ** Results taken from the comparative evaluation reported in [45]. NR = Not Reported.

Method	N-Caltech101			DVS128-Gesture		
	Param	Time-Step	Accuracy	Param	Time-Step	Accuracy
Spikformer [23]	2.57	16	83.6 **	2.57 *	16	98.30
SDT [24]	2.57	16	86.30 **	2.57 *	16	**99.30**
LSFormer [30]	NR	10	87.60 ± 0.10	NR	16	98.60 ± 0.30
TIM [46]	NR	10	79.00	NR	NR	NR
QKFormer [31]	1.57	16	87.24 **	1.5	16	98.60
Pairwise SDNA (ours)	1.95	16	88.95 ± 1.08	1.92	16	98.89 ± 0.38
Linear SDNA (ours)	1.95	16	**89.65 ± 0.61**	1.92	16	98.89 ± 0.29

**Table 5:** Average firing rates of the main spike-producing components in Pairwise and Linear SDNA on ImageNet. Values are reported as Stage 1/Stage 2/Stage 3.

Component	Pairwise SDNA	Linear SDNA
Q	0.1714/0.1757/0.2396	0.1321/0.1403/0.1030
K	0.3391/0.3146/0.1097	0.4302/0.4130/0.2995
V	0.1580/0.1443/0.0423	0.4220/0.4093/0.3015
Attention gate	0.0207/0.0175/0.0060	0.2967/0.2692/0.1352
SMLP Layer 1	0.0504/0.0285/0.0088	0.0494/0.0299/0.0098
SMLP Layer 2	0.3007/0.2748/0.2600	0.3005/0.2842/0.2665

**Table 6:** Accuracy (%) under a homogeneous membrane time constant τ on the event benchmarks. Bold numbers are the best performance.

τ	N-Caltech101		DVS128-Gesture	
	Pairwise	Linear	Pairwise	Linear
1.25	88.60	88.60	98.61	98.96
**1.50**	**89.18**	**89.47**	**99.31**	**99.31**
2.00	87.13	85.38	98.96	98.96
2.50	84.50	84.50	98.26	98.61
3.00	84.21	83.92	97.92	95.49

## Data Availability

All datasets in this study are publicly available and cited. The code implementing the proposed operators and reproducing the experiments is provided as Supplementary Material.

## Supplementary Materials

The following supporting information can be downloaded at: https://www.mdpi.com/article/10.3390/e28101130/s1. Supplementary Code S1: Implementation of Pairwise and Linear SDNA, including training and evaluation code for CIFAR-10, CIFAR-100, ImageNet, N-Caltech101, and DVS128-Gesture.

## Appendix A. Extended Method Section

### Appendix A.1. Spike Firing Approximation and Temporal Regimes

Binary firing emits at most one spike per step and cannot register how far the membrane exceeds the threshold, collapsing every activation to a single spike regardless of magnitude. The integer code of the Spike Firing Approximation (SFA) [32] preserves this magnitude and recovers representational precision, which mostly improves accuracy on dense inputs. During training, with the threshold set to unity, the neuron emits the integer

(A1) $${\mathrm{Fire}}_{D}\left(U\right)\;=\;\left\lfloor \mathrm{clip}\{U,\;0,\;D\}\right\rceil \;\in \;\left\{0,1,\ldots ,D\right\},$$

where clip{U,0,D} confines the membrane potential *U* to [0,D], ⌊·⌉ rounds to the nearest integer, and *D* is the maximum level, so a single step carries a graded value rather than one binary spike; the binary neuron is the case D=1. At inference the same integer is realized as a sequence of *D* binary spikes,

(A2) $$s\;=\;\sum_{d=1}^{D}\hat{S}\left[d\right],\;\hat{S}\left[d\right]\in \left\{0,1\right\},$$

so the network runs as a purely binary, spike-driven system while reproducing the integer used in training. The two are equal exactly under a soft-reset neuron at unit threshold, since a neuron given the membrane potential at the first sub-step d=1 and zero input thereafter fires once per sub-step and subtracts the threshold on firing, emitting precisely *s* spikes.

The model has two distinct time axes. The virtual sub-steps d=1,…,D above encode a single integer activation and are non-leaky, which is the condition that makes the integer-to-spike equivalence exact. Separately, the physical time-steps *t* carry the actual input sequence, and the two data modalities differ only in how the neuron behaves along this physical axis. On static images, the input is presented at a single physical step, so no membrane state is carried between steps and the integer level *D* alone provides the graded code. On neuromorphic data the input is a temporal sequence over several physical steps, and the membrane retains a decayed fraction β of its previous state between them (Equation (1)), so the neuron integrates evidence across the event stream while still emitting an integer per step. The decay is set by a time constant τ, a per-dataset setting reported with the results. Because the leak acts only across physical steps, τ has no effect on the static models, which run at a single physical step, and it is swept only for the event models.

### Appendix A.2. Locality, Kernel-Independence, and Spike-Drivenness

Global spike-driven attention is linear in *N* and spike-driven at once for a structural reason. It forms one aggregate, $\sum_{n}\left(K_{n}⊙V_{n}\right)$, and shares it across every query [24], so there is a single accumulation, nothing slides, and the cost is O(N). Locality removes the property that makes this possible, since a neighborhood operator needs a different window sum at every position and adjacent windows overlap as they slide across the feature map.

The usual way to compute overlapping window sums cheaply is an integral image. One precomputes a prefix sum and recovers any window in constant time by inclusion–exclusion, at O(1) per position and independent of *k*. We do not use it. Inclusion–exclusion subtracts dense, multi-bit prefix sums at every position, and subtraction of dense values is not a spike-gated accumulation, so the kernel-independent route is not spike-driven. The remaining option is to re-accumulate each window directly, which is spike-driven but costs $O(k^{2})$ per position and therefore depends on the kernel size. A local aggregator thus cannot be both kernel-independent and spike-driven under the two implementations one would actually reach for. We do not claim that no algorithm can attain all three properties, only that this trade is forced in practice, and we keep spike-drivenness.

### Appendix A.3. Inherited Backbone Components

The SPEDS module performs patch embedding, spikification, and inter-stage downsampling through a short stack of convolution, normalization and spiking-neuron units. The first SPEDS is the only place in the network that consumes real-valued input, and each subsequent SPEDS halves the spatial resolution and doubles the channel width. It uses max pooling rather than strided convolution, which on spike tensors preserves sparse activity, since a spike anywhere in the pooling footprint survives, rather than diluting it. The classification head reduces the final stage by global average pooling over space, averages over the *T* time-steps, and applies a single linear classifier; averaging over time after spatial pooling lets the readout integrate evidence across the temporal window, which matters on event data where individual time-steps are sparse.

## References

[1] Maass W. (1997). Networks of spiking neurons: The third generation of neural network models. Neural Netw..

[2] Kudithipudi D., Schuman C., Vineyard C.M., Pandit T., Merkel C., Kubendran R., Aimone J.B., Orchard G., Mayr C., Benosman R. (2025). Neuromorphic computing at scale. Nature.

[3] Karamimanesh M., Abiri E., Shahsavari M., Hassanli K., van Schaik A., Eshraghian J. (2025). Spiking neural networks on FPGA: A survey of methodologies and recent advancements. Neural Netw..

[4] Shahsavari M., Thomas D., van Gerven M., Brown A., Luk W. (2023). Advancements in spiking neural network communication and synchronization techniques for event-driven neuromorphic systems. Array.

[5] Roy K., Jaiswal A., Panda P. (2019). Towards spike-based machine intelligence with neuromorphic computing. Nature.

[6] Hill A.J., Donaldson J.W., Rothganger F.H., Vineyard C.M., Follett D.R., Follett P.L., Smith M.R., Verzi S.J., Severa W., Wang F. (2017). A spike-timing neuromorphic architecture. Proceedings of the 2017 IEEE International Conference on Rebooting Computing (ICRC).

[7] van Gerven M. (2026). Dynamical systems foundations for neuromorphic intelligence. Neuromorphic Comput. Eng..

[8] Tavanaei A., Ghodrati M., Kheradpisheh S.R., Masquelier T., Maida A. (2019). Deep learning in spiking neural networks. Neural Netw..

[9] Eshraghian J.K., Ward M., Neftci E.O., Wang X., Lenz G., Dwivedi G., Bennamoun M., Jeong D.S., Lu W.D. (2023). Training spiking neural networks using lessons from deep learning. Proc. IEEE.

[10] Fatahi M., Shahsavari M., Ahmadi M., Ahmadi A., Boulet P., Devienne P. (2018). Rate-coded DBN: An online strategy for spike-based deep belief networks. Biol. Inspired Cogn. Archit..

[11] Huebotter J., Lanillos P., van Gerven M., Thill S. (2026). Spiking neural networks for continuous control via end-to-end model-based learning. Neuromorphic Comput. Eng..

[12] Hassanshahi M., Shahsavari M., van Gerven M. (2024). Energy-efficient spiking recurrent neural network for gesture recognition on embedded GPUs. arXiv.

[13] Yin B., Corradi F., Bohté S.M. (2021). Accurate and efficient time-domain classification with adaptive spiking recurrent neural networks. Nat. Mach. Intell..

[14] Vaswani A., Shazeer N., Parmar N., Uszkoreit J., Jones L., Gomez A.N., Kaiser L., Polosukhin I. (2017). Attention is all you need. Proceedings of the 31st International Conference on Neural Information Processing Systems, Red Hook, NY, USA, 2017; NIPS’17.

[15] Devlin J., Chang M., Lee K., Toutanova K. (2019). BERT: Pre-training of deep bidirectional Transformers for language understanding. Proceedings of the North American Chapter of the Association for Computational Linguistics.

[16] Zhang H., Shafiq M.O. (2024). Survey of transformers and towards ensemble learning using transformers for natural language processing. J. Big Data.

[17] Dosovitskiy A., Beyer L., Kolesnikov A., Weissenborn D., Zhai X., Unterthiner T., Dehghani M., Minderer M., Heigold G., Gelly S. (2021). An image is worth 16x16 words: Transformers for image recognition at scale. Proceedings of the International Conference on Learning Representations.

[18] Li L.H., Yatskar M., Yin D., Hsieh C.J., Chang K.W. (2019). VisualBERT: A simple and performant baseline for vision and language. arXiv.

[19] Yuan L., Chen Y., Wang T., Yu W., Shi Y., Tay F.E.H., Feng J., Yan S. (2021). Tokens-to-token ViT: Training vision transformers from scratch on ImageNet. Proceedings of the IEEE/CVF International Conference on Computer Vision (ICCV).

[20] Carrigg K., van Gastel R., Yeghaian M., Dalm S., Boughorbel F., van Gerven M., Arai K., Lorenz P. (2026). Decorrelation speeds up vision Transformers. Proceedings of the Computer Vision Conference (CVC) 2026.

[21] Tay Y., Dehghani M., Bahri D., Metzler D. (2022). Efficient Transformers: A survey. ACM Comput. Surv. (CSUR).

[22] Carrigg K., de Vries S., Sadough A., van Gerven M. (2026). Evolving layer-specific scalar functions for hardware-aware transformer adaptation. arXiv.

[23] Zhou Z., Zhu Y., He C., Wang Y., YAN S., Tian Y., Yuan L. (2023). Spikformer: When spiking neural network meets Transformer. Proceedings of the Eleventh International Conference on Learning Representations.

[24] Yao M., Hu J., Zhou Z., Yuan L., Tian Y., Xu B., Li G. (2023). Spike-driven Transformer. Proceedings of the Thirty-Seventh Conference on Neural Information Processing Systems.

[25] Tang X., Chen T., Cheng Q., Shen H., Duan S., Wang L. (2025). Spatio-temporal channel attention and membrane potential modulation for efficient spiking neural network. Eng. Appl. Artif. Intell..

[26] Zhou S., Yang B., Yuan M., Jiang R., Yan R., Pan G., Tang H. (2024). Enhancing SNN-based spatio-temporal learning: A benchmark dataset and cross-modality attention model. Neural Netw..

[27] Hassani A., Walton S., Li J., Li S., Shi H. (2023). Neighborhood attention transformer. Proceedings of the IEEE/CVF Conference on Computer Vision and Pattern Recognition.

[28] Liu Z., Lin Y., Cao Y., Hu H., Wei Y., Zhang Z., Lin S., Guo B. (2021). Swin Transformer: Hierarchical vision transformer using shifted windows. Proceedings of the IEEE/CVF International Conference on Computer Vision.

[29] Jiao J., Tang Y.M., Lin K.Y., Gao Y., Ma A.J., Wang Y., Zheng W.S. (2023). DilateFormer: Multi-scale dilated transformer for visual recognition. IEEE Trans. Multimed..

[30] Li L., Zhang H., Yu Q. (2026). Breaking global self-attention bottlenecks in Transformer-based spiking neural networks with local structure-aware self-attention. arXiv.

[31] Zhang H., Zhou Z., Yu L., Huang L., Fan X., Yuan L., Ma Z., Zhou H., Tian Y. (2024). QKFormer: Hierarchical spiking transformer using Q-K attention. Proceedings of the Advances in Neural Information Processing Systems (NeurIPS).

[32] Yao M., Qiu X., Hu T., Hu J., Chou Y., Tian K., Liao J., Leng L., Xu B., Li G. (2025). Scaling spike-driven Transformer with efficient spike firing approximation training. IEEE Trans. Pattern Anal. Mach. Intell..

[33] Shaw P., Uszkoreit J., Vaswani A. (2018). Self-attention with relative position representations. Proceedings of the 2018 Conference of the North American Chapter of the Association for Computational Linguistics: Human Language Technologies.

[34] Chu X., Tian Z., Zhang B., Wang X., Shen C. (2023). Conditional positional encodings for vision Transformers. Proceedings of the Eleventh International Conference on Learning Representations.

[35] Krizhevsky A. (2009). Learning Multiple Layers of Features from Tiny Images.

[36] Deng J., Dong W., Socher R., Li L.J., Li K., Fei-Fei L. (2009). ImageNet: A large-scale hierarchical image database. Proceedings of the 2009 IEEE Conference on Computer Vision and Pattern Recognition.

[37] Orchard G., Jayawant A., Cohen G.K., Thakor N. (2015). Converting static image datasets to spiking neuromorphic datasets using saccades. Front. Neurosci..

[38] Amir A., Taba B., Berg D., Melano T., McKinstry J., Di Nolfo C., Nayak T., Andreopoulos A., Garreau G., Mendoza M. (2017). A low power, fully event-based gesture recognition system. Proceedings of the 2017 IEEE Conference on Computer Vision and Pattern Recognition (CVPR).

[39] Lee D., Li Y., Kim Y., Xiao S., Panda P. (2025). Spiking Transformer with spatial-temporal attention. Proceedings of the 2025 IEEE/CVF Conference on Computer Vision and Pattern Recognition (CVPR).

[40] Fang Y., Wang Z., Zhang L., Cao J., Chen H., Xu R. (2024). Spiking wavelet transformer. Proceedings of the European Conference on Computer Vision.

[41] Zhang H., Sboev A., Rybka R., Yu Q. (2025). Combining aggregated attention and transformer architecture for accurate and efficient performance of spiking neural networks. Neural Netw..

[42] Deng S., Wu Y., Du K., Gu S. (2024). Spiking token mixer: An event-driven friendly Former structure for spiking neural networks. Proceedings of the Advances in Neural Information Processing Systems.

[43] Cubuk E.D., Zoph B., Shlens J., Le Q. (2020). Randaugment: Practical automated data augmentation with a reduced search space. Adv. Neural Inf. Process. Syst..

[44] Zhong Z., Zheng L., Kang G., Li S., Yang Y. (2020). Random erasing data augmentation. Proceedings of the AAAI Conference on Artificial Intelligence.

[45] Zheng Z., Huang Y., Yu Y., Zhu Z., Tang J., Yu Z., Jin Y. (2025). Spiliformer: Enhancing spiking transformers with lateral inhibition. Proceedings of the 2025 IEEE/CVF International Conference on Computer Vision (ICCV).

[46] Shen S., Zhao D., Shen G., Zeng Y. (2024). TIM: An efficient temporal interaction module for spiking transformer. Proceedings of the Thirty-Third International Joint Conference on Artificial Intelligence, IJCAI’24.

[47] Horowitz M. (2014). 1.1 computing’s energy problem (and what we can do about it). Proceedings of the 2014 IEEE International Solid-State Circuits Conference Digest of Technical Papers (ISSCC).

